# Path Learning in Individuals With Down Syndrome: The Challenge of Learning Condition and Cognitive Abilities

**DOI:** 10.3389/fpsyg.2021.643702

**Published:** 2021-03-25

**Authors:** Chiara Meneghetti, Enrico Toffalini, Silvia Lanfranchi, Maja Roch, Barbara Carretti

**Affiliations:** ^1^Department of General Psychology, University of Padova, Padua, Italy; ^2^Department of Developmental Psychology and Socialization, University of Padova, Padua, Italy

**Keywords:** down syndrome, route learning, floor matrix task, visuospatial abilities, working memory, observation, oral instructions

## Abstract

Analyzing navigational abilities and related aspects in individuals with Down syndrome (DS) is of considerable interest because of its relevance to everyday life. This study investigates path learning, the conditions favoring it, and the cognitive abilities involved. A group of 30 adults with DS and 32 typically-developing (TD) children matched on receptive vocabulary were shown a 4 × 4 Floor Matrix and asked to repeat increasingly long sequences of steps by walking on the grid. The sequences were presented under two learning conditions, one called Oral instructions (participants received verbal instructions such as “turn right” or “turn left”), the other Observation (participants watched the experimenter's moves). Participants were also assessed on verbal and visuospatial cognitive measures. The results showed a similarly better performance in both groups when the Floor Matrix task was administered in the Observation as opposed to the Oral instructions condition. As for the relation with cognitive abilities, in the Floor Matrix task in the Oral instructions condition, individuals with DS showed an effect of both verbal and visuospatial abilities, which was only positive for verbal ability. The effect of verbal and visuospatial abilities was negligible in the TD group. In the Observation condition, performance was predicted by sequential working memory in both groups. Overall, these results shed light on path learning in individuals with DS, showing that they benefited from the Observation condition, and that the involvement of their cognitive abilities depended on the learning condition.

## Introduction

Being able to move around in the environment is essential to daily living. It entails a complex process of memorizing sequences of movements and changes of direction, experienced from an egocentric point of view, based on sensorimotor (e.g., vestibular and kinesthetic) information Montello (2005). The way in which spatial information is conveyed affects our navigation ability. For instance, we may receive verbal instructions from somebody about how to reach a given place, or we may copy someone else's moves. Either way, we form a spatial mental representation (or cognitive map; Tolman, 1948), but the type of information used to do so can influence its final features (e.g., Picucci et al., 2013).

The source of spatial information can be considered as the initial level of our environment learning ability. Already at this level, it is worth examining individual differences in performance and the cognitive abilities involved (Meneghetti et al., 2020c). In the present paper, we focus on individuals with Down syndrome (DS), a condition caused by chromosome 21 trisomy. Individuals with DS generally have an intelligence quotient between 25 and 70, and a mental age of 5–7 years (Dykens et al., 2000; Kittler et al., 2008). They are generally recognized as having relatively stronger visuospatial than verbal abilities (Dykens et al., 2000; Silverman, 2007). Visuospatial abilities include a number of skills, however, and a given individual's profile may have peaks and troughs (Yang et al., 2014). Some studies have focused on the ability to retain and process spatial information, i.e., working memory (WM). Individuals with DS have been found to perform better in sequential WM tasks (remembering increasingly long sequences) than in simultaneous WM tasks (recalling increasingly numerous locations). It is only in sequential WM that the performance of individuals with DS is comparable with typically-developing (TD) children matched on cognitive functioning and therefore in line with their mental age (Lanfranchi et al., 2004; Carretti and Lanfranchi, 2010).

### Path Learning and Visual Input

Navigation and related aspects have been little examined so far in individuals with DS (Yang et al., 2014; Meneghetti et al., 2019), despite the importance for this population of being able to explore the environment and move around to reach places (Yang et al., 2018). The available literature (Courbois et al., 2013; Davis et al., 2014; Farran et al., 2015; Purser et al., 2015; Toffalini et al., 2018; Himmelberger et al., 2020) suggests that, after exploring (mostly in virtual environments), individuals with DS can follow a path they have seen, which demands egocentrically-presented knowledge (i.e., based on self-to-object relations). They find it more difficult, however, to reorganize their information allocentrically—to find a shorter route, for instance (i.e., based on object-to-object relations). More simple, regular environments seem to facilitate the recall of object-to-object relations (Himmelberger et al., 2020).

If individuals with DS handle environmental information from an egocentric perspective better than from an allocentric one, it is worth investigating how much egocentrically-presented sequential information they can learn and retrieve. This can be examined better by considering actual body movements. Some inspiring evidence comes from examining TD children's ability to learn increasingly long sequences in vista space (i.e., a fully-visible space in which a person can move; Montello, 1998). Piccardi et al. (2008) tested children's ability to learn increasingly long paths using the Walking Corsi task, a large-scale version of the classical Corsi Blocks task (a board with nine irregularly-placed blocks; Corsi, 1973) in a vista space setting. They found this ability increases with age, and TD children around 5–7 years old (which roughly corresponds to the mental age of individuals with DS) could remember about three squares (Piccardi et al., 2014a,b, 2015). Being egocentrically based, this approach could also shed light on the case of individuals with DS. Meneghetti et al. (2020b) recently compared individuals with DS and TD children matched on cognitive functioning on their ability in the Floor Matrix task, which involves learning increasingly long sequences of steps on a 4 × 4 matrix (2.3 × 2.3 m) in a regular-shaped grid. The sequence was either demonstrated by the experimenter (observation condition) or shown on a map (map condition), as even children 3–5 years old understand the representative function of maps (Frick and Newcombe, 2012). The results showed that both groups were able to learn the sequences, and both performed better in the observation condition than in the map condition, and to much the same extent. Taken together, studies conducted to date show that individuals with DS can acquire spatial information from a person (egocentric) point of view from visual sources such as watching someone move or a video of a path.

### Path Learning and Verbal Input

Another way to receive spatial information from an egocentric perspective is to listen to spoken instructions. This demands verbal abilities. Individuals with DS have more difficulty with productive than with receptive vocabulary (Chapman and Hesketh, 2000), and with abstract than with concrete words (Lorusso et al., 2017). They also have trouble understanding complex sentences (Levorato et al., 2009; Frizelle et al., 2019) and texts, especially when conveyed orally, by comparison with their reading comprehension (Roch et al., 2012, 2019). It is important to note that the content of instructions conveys spatial information. The literature on typical development shows that the use of spatial language is related to performance in spatial tasks already at 4–5 years of age (Pruden et al., 2011). As regards spatial descriptions, preschoolers (4–5-year-olds) have proved capable of managing descriptions from the person's point of view (self-to-object relations) and from map view (object-to-object relations) (Nyhout and O'Neill, 2013; Meneghetti et al., 2020a). There is evidence of individuals with DS understanding spatial relations (e.g., left/right sides of objects) and learning spatial information from the person's or map view (Meneghetti et al., 2017). In other words, they can manage spatial descriptions despite their difficulty in managing linguistic information. Since the TD literature shows that spatial language is associated with visuospatial abilities, it seems worth examining whether individuals with DS can manage egocentrically-presented spatial information when it is conveyed linguistically, as well as visually.

### Path Learning and Cognitive Abilities

Environment learning is known to relate to visuospatial abilities (Hegarty et al., 2006) already at 5–6 years old (e.g., Purser et al., 2012, 2015; Merrill et al., 2016; Thomas et al., 2016). For example, 6-year-olds' performance in the Walking Corsi Test (WalCT) was found related to individual visuospatial skills, such as field-independent cognitive style (Boccia et al., 2019); and, when the squares were identified using images of landmarks, performance related to verbal abilities, such as grammar comprehension (Piccardi et al., 2015). The few studies on individuals with DS (Davis et al., 2014; Farran et al., 2015; Purser et al., 2015) found that their environment learning (especially in reproducing a path) was related to their visuospatial reasoning [measured with Raven's colored progressive matrixes [CPM; Raven et al., 1998]] and other cognitive abilities (executive control, attention and memory). Visuospatial reasoning seems crucial to path learning, especially in individuals with DS (Farran et al., 2015; Purser et al., 2015). There is also a link between the learning of actual moves and visuospatial abilities. Meneghetti et al. (2020b) found that visuospatial abilities like mental rotation and spatial WM predicted performance in a Floor Matrix task, and more strongly in the observation than in the map condition. This aroused our interest in clarifying the role of a set of cognitive abilities in supporting path learning by individuals with DS.

### Rationale and Aim of the Study

The aims of the present study were to compare individuals with DS with TD children matched on cognitive functioning regarding: (i) the ability to learn actual paths of increasing length in different conditions (from observation or oral instructions); and (ii) how their performance in these two path learning conditions related to their cognitive abilities. The two groups were administered a Floor Matrix task (as in Meneghetti et al., 2020b). They watched a person move through a sequence of squares (as in Piccardi et al., 2008) or were given oral instructions using egocentric language that individuals with DS have proved able to understand (Meneghetti et al., 2017). Then they repeated the sequence of steps. Verbal and visuospatial measures were administered as proxies of general cognitive abilities. Sequential WM and mental rotation abilities were also assessed.

We expected to find both groups capable of performing the Floor Matrix task to some degree. Some differences between learning conditions were expected, with oral instructions proving more difficult to learn from than observation. This could be particularly true of individuals with DS, given their greater weakness in verbal abilities (Lorusso et al., 2017; Roch et al., 2019) than in visuospatial abilities (Silverman, 2007). Alternatively, the difference vis-à-vis matched TD children might be smaller if the spatial content (which individuals with DS can understand; Meneghetti et al., 2017) prevails even though the information is presented verbally. We also expected participants' cognitive abilities to influence their performance in the Floor Matrix task, here again as a function of learning condition. Learning a path from observation (a non-verbal condition) should mainly demand visuospatial abilities, with sequential WM likely to have a central role, given the involvement of abilities similar to those required for the Floor Matrix task (i.e., sequences of increasing length; Meneghetti et al., 2020b). Learning from oral instructions should engage both verbal abilities, given the format used to deliver the information, and visuospatial abilities, given its content (Piccardi et al., 2015; Meneghetti et al., 2017).

### Participants

The sample consisted of two groups: 30 individuals with DS (mean age = 30.26 years, SD = 9.22, range 21.0–53.3 years, 18 females), and 32 TD children (mean age = 5.69 years, SD = 1.12, range 3.2–8.0 years, 11 females). The two groups were matched on two typical measures of intellectual functioning. A measure of receptive vocabulary, the Peabody Picture Vocabulary Test—Revised (Dunn and Dunn, 1997; Italian adaptation by Stella et al., 2000; PPVT-R), was used as a verbal task. It involves indicating which of four black-and-white drawings best represents a word spoken by the experimenter; and there are 175 pictorial stimuli of increasing difficulty. The final score is the total number of drawings correctly identified (range 0–175). Raven's Colored Progressive Matrices (Raven et al., 1998; Italian adaptation by Belacchi et al., 2008; CPM) was used as a visuospatial task. Respondents choose one of six pieces that best fits the piece that is missing from a matrix; and there are 36 increasingly complex matrices. The final score is the number of matrices correctly completed (range 0–36). The two groups were well-matched on the PPVT-R, t(60) = −0.25, *p* = 0.80, Cohen's d = −0.06, but less so on the CPM, with average scores moderately lower in the DS group than in the TD group, t(60) = −1.98, *p* = 0.05, Cohen's d = −0.50.

### Materials

#### Floor Matrix Task (Adapted From Meneghetti et al., 2020b)

A 4 × 4 matrix on the floor consists of 16 squares (of stiff cardboard), each 50 × 50 cm in size, placed with a 10 cm gap between them to form a square layout covering ~2.3 × 2.3 m. The task involves receiving information about sequences of positions in the matrix and then reproducing the sequences in the same order. The information was presented in two conditions: in one, participants watched the examiner make a series of moves to complete the sequence (Observation); in the other, they were given verbal indications (Oral instructions). In the Observation condition, the experimenter stood in the square marked with an “X,” then completed a sequence of steps, stopping for 3 s in each square, while a participant stood outside the matrix in front of square “X” and watched the experimenter's moves. In the Oral instructions condition, participants stood in square “X” and the experimenter stood next to them, giving spoken egocentric directions, one every 3 s (“turn right and take a step,” “turn left and take a step,” or “take a step forward”). Two parallel versions of the task were prepared and counterbalanced across conditions. The layouts of the matrix with the paths for all the trials are shown in Supplementary Figure 1. Immediately after a sequence had been presented, the participant which was asked to reproduce exactly the same sequence of steps by walking in the matrix, covering the path they had observed or heard described. Two trials for each condition (with 2-step sequences) were used for familiarization purposes, and the right moves were shown if participants made mistakes. There were 10 trials for each learning condition, two for each sequence with the same number of steps (which ranged from 1 to 5). The task was terminated when a participant failed to complete both trials with the same number of steps. The final score was the number of correctly completed trials (range 0–10).

#### Individual Visuospatial Measures

##### Ghost Picture Test (GPT; Adapted From Frick et al., 2013)

This test consists of 21 items, each depicting a target silhouette of a ghost inside a circle at the top of the page. Below, there are two similar silhouettes and respondents must choose which of the two is identical to the target figure in a rotated position (the other is a mirror image). The items require different degrees of rotation to match the target figure (from 0° to 180°). The final score was the total number of correct answers (range 0–21). The internal consistency was good, 0.83 (current sample).

##### Working Memory Matrices—Sequential (WMM-S; Lanfranchi et al., 2004)

Square matrices comprising 3 × 3 and 4 × 4 cells (each cell measuring 3 cm) are presented on a sheet of paper. The experimenter shows a path covered on the matrix by a small frog, which jumps onto some of the cells in the matrix, stopping at each cell for 1 s. Participants were asked to reproduce the sequences of jumps immediately afterwards in the right order. There were 8 trials of increasing difficulty, deriving from the size of the matrix and the number of jumps to remember (from 2 to 4). The final score was the number of trials completed correctly (range 0–8). The internal consistency is moderately good (0.59; Lanfranchi et al., 2004).

### Procedure

In a preliminary session, after parents (or legal representatives) and individuals with DS over 18 gave their consent, participants individually completed the PPVT-R and CPM. Then they were tested individually during two further sessions on two different days in the same week (for the participants' convenience) at their day center or school. The Floor Matrix task was administered in the first session (the order of learning condition was balanced across participants). Before proceeding, the experimenter ascertained that participants identified the right and left sides of their body correctly. The CMP and WMM-S were administered in the second session, in counterbalanced order. Specific instructions were given for each measure, making sure participants understood the task by practicing first with examples.

## Results

Table 1 shows the descriptive statistics (means, standard deviations), and Table 2 shows the correlations, for the two groups, for all variables of interest. Scatterplots and histograms are shown in Supplementary Figures 2–4.

**Table 1:** Means (M) and standard deviations (SD) for individuals with DS (*N* = 30) and typically-developing (TD) children (*N* = 32).

**Variable**	**DS group**		**TD group**	
	**M**	**SD**	**M**	**SD**
1. Floor matrix task (oral instructions)	2.50	1.07	2.44	1.85
2. Floor matrix task (observation)	6.87	1.81	7.19	2.72
3. PPVT-R	73.07	30.70	74.97	29.58
4. CPM	14.87	4.25	17.62	6.42
5. WMM-S	5.30	1.97	4.62	1.77
6. GPT	13.07	3.13	15.00	4.58

*For descriptive purposes, we also calculated the span scores: Observation condition: DS: M = 3.83, SD = 0.99; TD: M = 4.03, SD = 1.45; Oral condition: DS: M = 1.43, SD = 0.68; TD: M = 1.59, SD = 1.13). PPVT-R, Peabody Picture Vocabulary Test-Revised; CPM, Raven's Colored Progressive Matrices; WMM-S, Working Memory Matrices-Sequential; GPT, Ghost Picture Test*.

**Table 2:** Correlations with confidence intervals for individuals with DS (below diagonal, *N* = 30), and typically-developing (TD) children (above diagonal, *N* = 32).

**Variable**	**1**	**2**	**3**	**4**	**5**	**6**
1. Floor matrix task	-	0.31	−0.01	0.32	−0.07	0.22
(oral instructions)		[−0.04, 0.60]	[−0.36, 0.34]	[−0.03.60]	[−0.41, 0.29]	[−0.14, 0.52]
2. Floor matrix task	0.19	-	0.03	0.10	0.44*	0.28
(observation)	[−0.18, 0.52]		[−0.32, 0.37]	[−0.25, 0.44]	[0.11, 0.68]	[−0.08, 0.57]
3. PPVT-R	0.44*	0.50**	-	0.47**	0.12	0.02
	[0.09, 0.69]	[0.17, 0.73]		[0.14, 0.70]	[−0.24, 0.45]	[−0.33, 0.37]
4. CPM	−0.36*	0.33	0.41*	–	0.21	0.18
	[−0.64, −0.00]	[−0.04, 0.62]	[0.06, 0.67]		[−0.15, 0.52]	[−0.18, 0.50]
5. WMM-S	−0.02	0.68**	0.43*	0.50**	-	−0.06
	[−0.38, 0.34]	[0.42, 0.83]	[0.08, 0.68]	[0.17, 0.73]		[−0.40, 0.30]
6. GPT	−0.05	0.31	0.27	0.43*	0.13	–
	[−0.40, 0.31]	[−0.06, 0.60]	[−0.10, 0.57]	[0.08, 0.68]	[−0.25, 0.46]	

Values in square brackets indicate 95% confidence intervals for each correlation.

* indicates p < 0.05;

** indicates p < 0.01. PPVT-R, Peabody Picture Vocabulary Test-Revised; CPM, Raven's Colored Progressive Matrices; WMM-S, Working Memory Matrices-Sequential; GPT, Ghost Picture Test.

*For TD children, correlations are partialized by chronological age*.

Importantly, since the age range of the TD group was wide (3.2–8.0 years), and the children were all of developmental age, correlations for the TD group were partialized by age. The bivariate correlations between all variables and age were all r ≥ 0.75 in the TD group, meaning that any uncorrected relation would be strongly inflated. The same correction was not applied for the DS group because all the participants with DS were adults. All correlations were found generally stronger in the DS group than in the TD group (see Table 2 for details).

### Path Learning: the Effect of Learning Condition (Observation vs. Oral Instructions)

To examine the effect of learning condition on performance, and whether its effect differed in the two groups (our first aim), a 2 (condition: oral instructions vs. observation, within participants) × 2 (group: DS vs. TD, between participants) mixed ANOVA was run on participants' scores. A significant main effect of condition emerged, *F*_(1, 60)_ = 366.01, *p* < 0.001, such that performance was much better in the observation than in the oral instructions condition, Cohen's d = 2.34 [95% CI: 1.88, 2.80]. There was no main effect of group, *F*_(1, 60)_ = 0.09, *p* = 0.77 (the between-group difference in performance was negligible, Cohen's d = 0.04 [−0.40, 0.31]]. Nor was there any significant interaction between condition and group, *F*_(1, 60)_ = 0.65, *p* = 0.42, and this was shown by the fact that the effect of condition was large and similar in the two groups: for DS, Cohen's d = 2.93 [2.18, 3.67]; for TD, Cohen's d = 2.04 [1.43, 2.66].

### Path Learning: The Effect of Individual Verbal and Visuospatial Abilities

Linear models were used to examine how well-cognitive variables could predict performance in the Floor Matrix task, as a function of learning condition and group (our second aim). This was done by calculating the *R*^2^ of different linear models in which the cognitive variables were entered as predictors, and scores in the Floor Matrix task as dependent variables. A model selection procedure was adopted to avoid *R*^2^ being inflated due to several predictors being entered at the same time. Specifically, the best-fitting combination, i.e., the one that minimized the Akaike Information Criterion (AIC; Akaike, 1974), was selected. To establish the confidence intervals (CIs) of the *R*^2^ estimate, the final best-fitting models were bootstrapped for 10,000 iterations. The 95% CIs were calculated with the percentile method.

Separate linear models were fitted by condition and by group, both for the sake of simplicity in calculating the *R*^2^, and because the two versions of the task were expected to pose different demands, and thus have different sets of predictors.

#### Floor Matrix Task—Oral Instructions

In the DS group, the best-fitting model included the CPM and PPVT-R as predictors, and both were significant (*p*s < 0.001), though the former had a negative coefficient. The variance explained was high, *R*^2^ = 0.55 [95% CI: 0.28, 0.76]. In the TD group, the best-fitting model included only the CPM, which failed to reach the significance threshold, *p* = 0.08. The variance explained was low, *R*^2^ = 0.10 [0.00, 0.38].

#### Floor Matrix Task—Observation

In the DS group, the best-fitting model included the PPVT-R, GPT, and WMM-S as predictors. Only the association with the WMM-S was positive (*p* < 0.001), while neither the PPVT-R (*p* = 0.18) nor the GPT (*p* = 0.19) were very relevant. The total variance explained was high, however, *R*^2^ = 0.55 [95% CI: 0.35, 0.80]. In the TD group, the best-fitting model included only the GPT and WMM-S. Once again, only the WMM-S had a significant role, with a positive coefficient (*p* = 0.007), while the GPT failed to reach the conventional significance threshold (*p* = 0.06). The variance explained was moderate, *R*^2^ = 0.29 [0.09, 0.55].

## Discussion

The impact of learning condition on path learning in the Floor Matrix task (aim 1) showed that performance was better in the observation than in the oral instructions condition, in both groups. In the observation condition, both groups remembered sequences involving 3–4 steps, on average. These results are consistent with previous studies assessing individuals with DS on sequences of moves in vista space (with the Floor Matrix task; Meneghetti et al., 2020b) and TD 5- to 6-year-olds (with the Walking Corsi test; Piccardi et al., 2014a). In the oral instructions condition, not examined in previous studies, both groups performed poorly in the Floor Matrix task. In short, individuals with DS and TD children on a similar cognitive functioning level learn a path better from watching actual moves than from spoken instructions. The advantage of observation might stem from the consistency between the learning and reproducing modalities (path sequences presented and reproduced visuospatially), whereas the change of format in the oral instructions condition (from a language-based presentation to a visuospatial reproduction) poses more problems.

The difference between observation and oral instructions emerges better on analyzing the contribution of cognitive abilities to performance in the Floor Matrix task (aim 2). In the observation condition, a specific role of sequential WM emerged in both groups. In the oral instructions condition, the DS group's performance was predicted by both their verbal abilities (receptive vocabulary, with a positive association) and their visuospatial abilities (reasoning, with a negative association), whereas the role of these abilities was marginal in the TD group. The amount of variance in accounting for performance was also larger in the DS than in the TD group (as previously reported by Farran et al., 2015).

The different involvement of cognitive abilities as a function of learning condition and group deserves further consideration. Intriguingly, the TD and DS groups' performance was similar in the oral instructions condition, but the cognitive abilities involved differed. In individuals with DS, there was a clear influence of both verbal and visuospatial abilities on their Floor Matrix task performance. Though this is consistent with evidence of a role for TD children's verbal abilities in a vista space task (e.g., grammar comprehension in the Walking Corsi test; Piccardi et al., 2015), individuals with DS have weak verbal skills, struggling to understand orally-presented abstract sentences, for instance (Levorato et al., 2009; Frizelle et al., 2019) -though they can understand orally-presented spatial relations (Meneghetti et al., 2017). The verbal skills (receptive vocabulary) of our individuals with DS became relevant in predicting their moves prompted by oral instructions. In other words, individuals with DS with a certain level of receptive vocabulary were able to accomplish the task, which involves understanding verbally-conveyed instructions (e.g., “turn right and take a step”). They found it very verbal-resource-consuming. This prevented them from translating the information into a visuospatial mental representation (in order to reproduce the moves in the matrix). In this situation, recruiting visuospatial abilities (such as visuospatial reasoning) might be detrimental to performance in the Floor Matrix task. These findings are also consistent with the difficulties individuals with DS encounter in processing and switching information (e.g., Lanfranchi et al., 2012). It thus seems that the contribution of cognitive abilities differed between our two groups, and that a learning condition involving oral instructions on how to approach a spatial task (based on sequences of moves) demands a mix of cognitive abilities in individuals with DS. This issue is worth examining in further studies.

The role of sequential WM emerged in the observation condition. This shows that a small-scale sequential WM task demands the same resources as a Floor Matrix task (i.e., in vista space), supporting the idea of the latter as a navigational WM task (Piccardi et al., 2015). Previous studies also found spatial WM involved, as well as visuospatial abilities, such as rotation (Meneghetti et al., 2020b) and visuospatial reasoning (Farran et al., 2015). This difference may depend on the type of task (large-scale in Farran et al., 2015; vista space in the present study), and the age of individuals with DS (who were younger in Meneghetti et al., 2020b).

## Conclusion

Overall, this study contributes to expanding what we know about path learning in a controlled setting in individuals with DS, showing which condition facilitates it and the abilities involved. Individuals with DS can learn increasingly-long sequences of steps just as well as TD children matched on cognitive functioning. They clearly benefit from being shown a path by a person (from direct observation), with a specific involvement of sequential WM. Their performance is worse when spatial information is given using oral instructions, revealing a differential involvement of their verbal and visuospatial abilities.

## Data Availability Statement

The datasets presented in this study can be found in online repositories. The names of the repository/repositories and accession number(s) can be found below: https://doi.org/10.6084/m9.figshare.13379630.

## Ethics Statement

The studies involving human participants were reviewed and approved by the Ethics Committee for Research in Psychology at the University of Padova (N. 3519). Written informed consent to participate in this study was provided by the participants' legal guardian/next of kin.

## Author Contributions

ET performed the statistical analysis. ET and CM organized the database. CM wrote the first draft of the manuscript. All authors contributed to sections of the manuscript, to revising the manuscript, they read and approved the submitted version, and conception and design of the study.

## Conflict of Interest

The authors declare that the research was conducted in the absence of any commercial or financial relationships that could be construed as a potential conflict of interest.
