# Supplementary material for: Path Learning in Individuals With Down Syndrome: The Challenge of Learning Condition and Cognitive Abilities
Source: Front Psychol. 2021 Mar 25;12:643702. doi: 10.3389/fpsyg.2021.643702 (PMC8027337; doi:10.3389/fpsyg.2021.643702)
Supplement: Supplementary file 1 [file Data_Sheet_1.PDF]

Supplementary Material

1 Supplementary figures

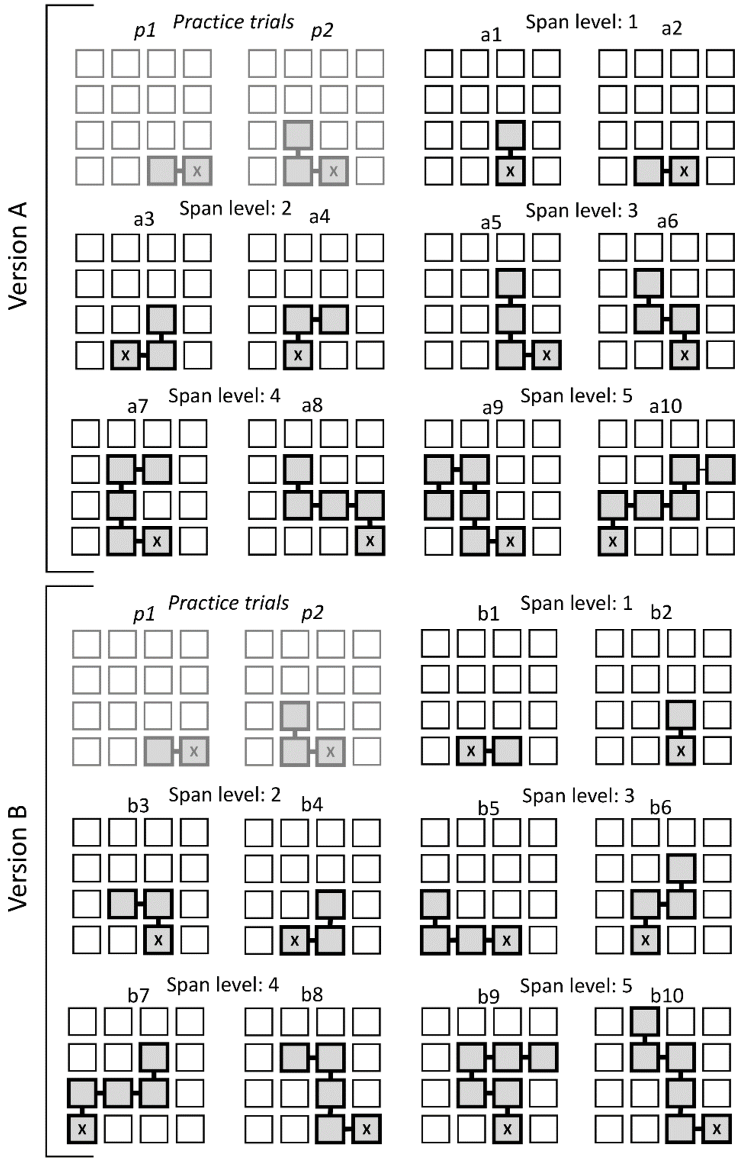

Supplementary Figure 1. Layouts of the 4x4 Floor Matrix stimuli with all the paths to be learnt in both versions of the task. The square marked with an "X" represents the starting point on each path. In the "oral instructions" condition the instructions are given with a series of sentences ("turn right and take a step", "turn left and take a step", or "take a step forward").

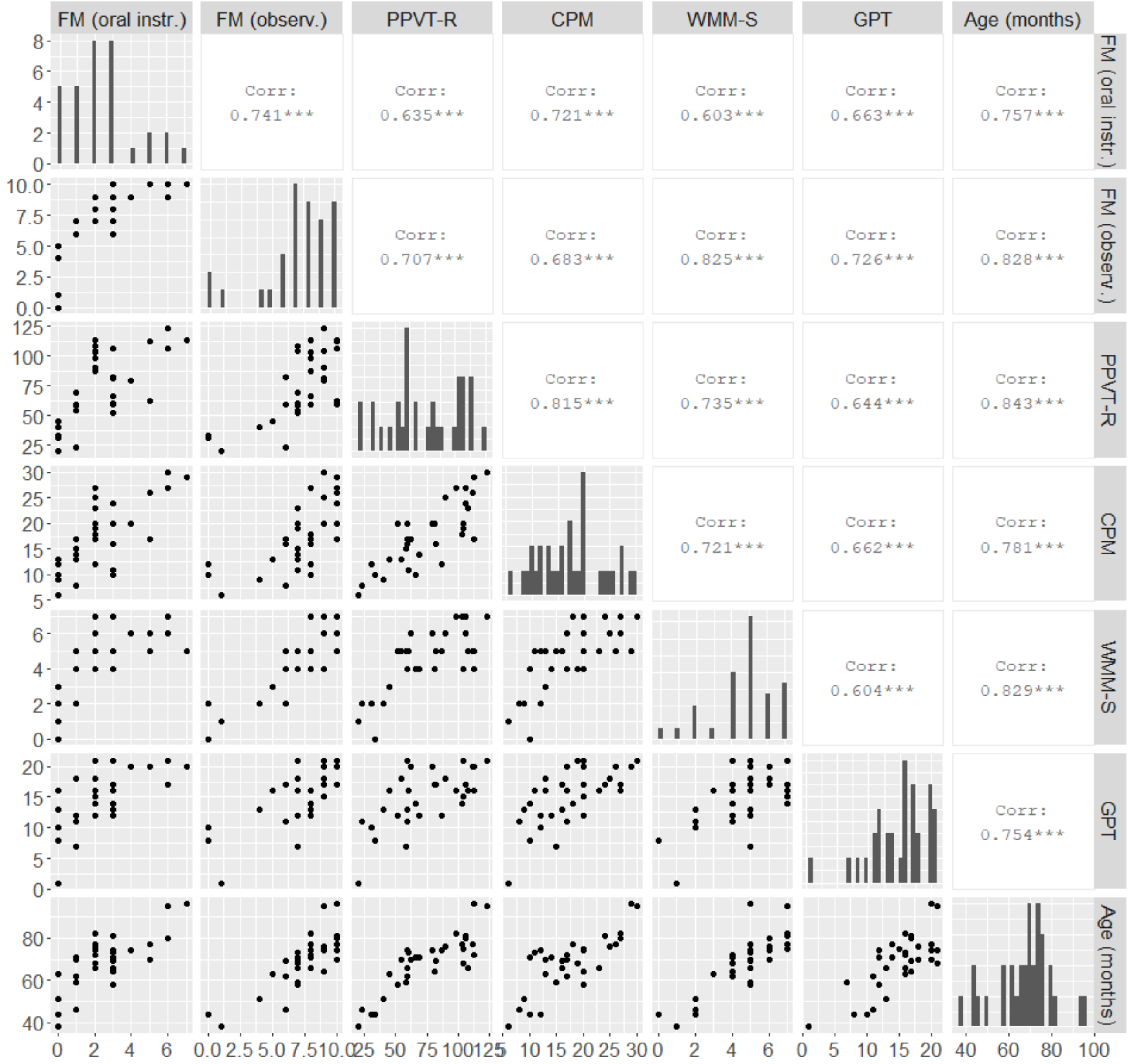

Supplementary Figure 2. Scatterplots, histograms, and correlations for all variables of interest and chronological age (in months) of participants in the typically-developing group (N = 32).

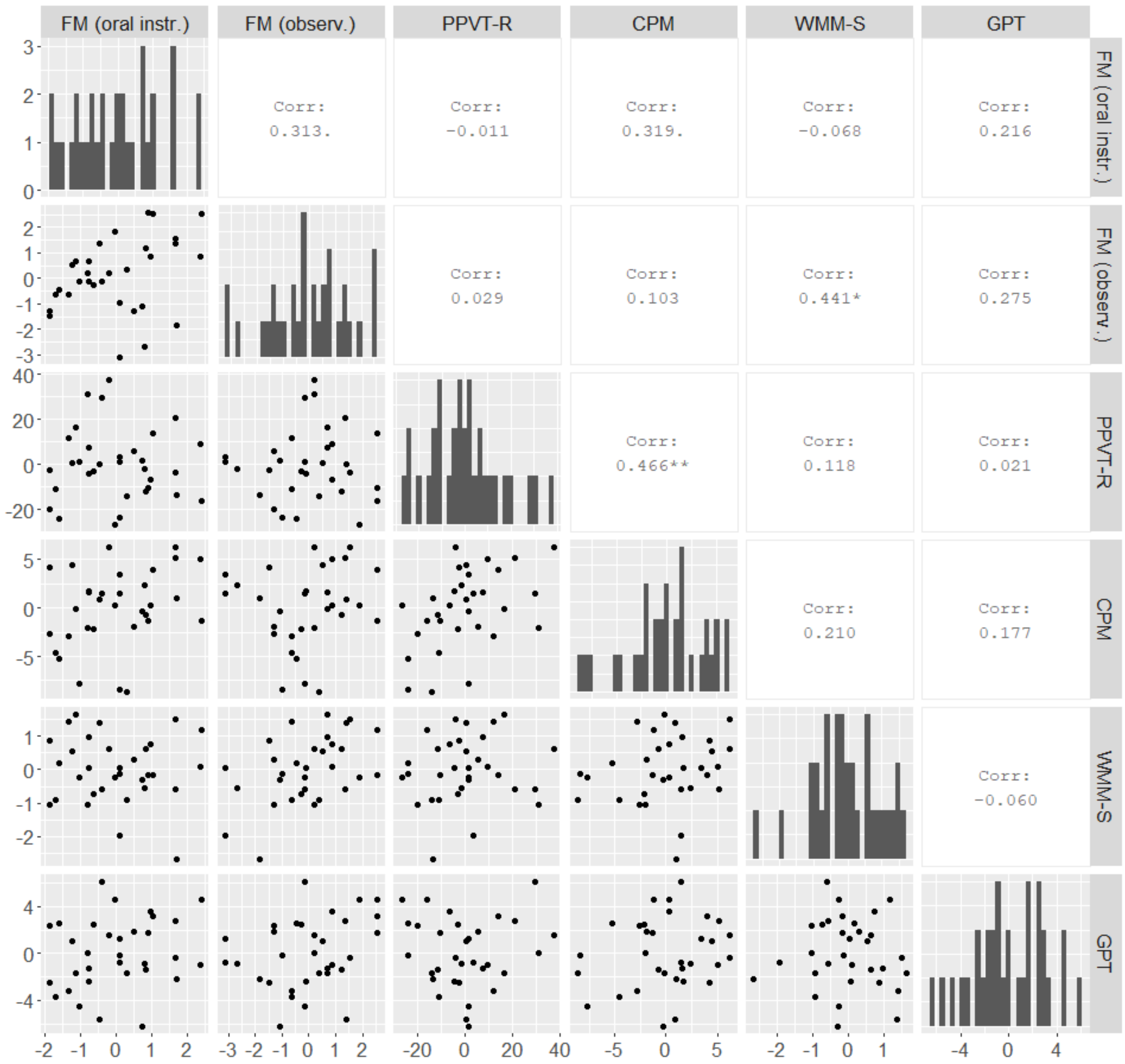

Supplementary Figure 3. Scatterplots, histograms, and correlations for all variables of interest, residualized by chronological age (in months) of participants in the typically-developing group (N = 32).

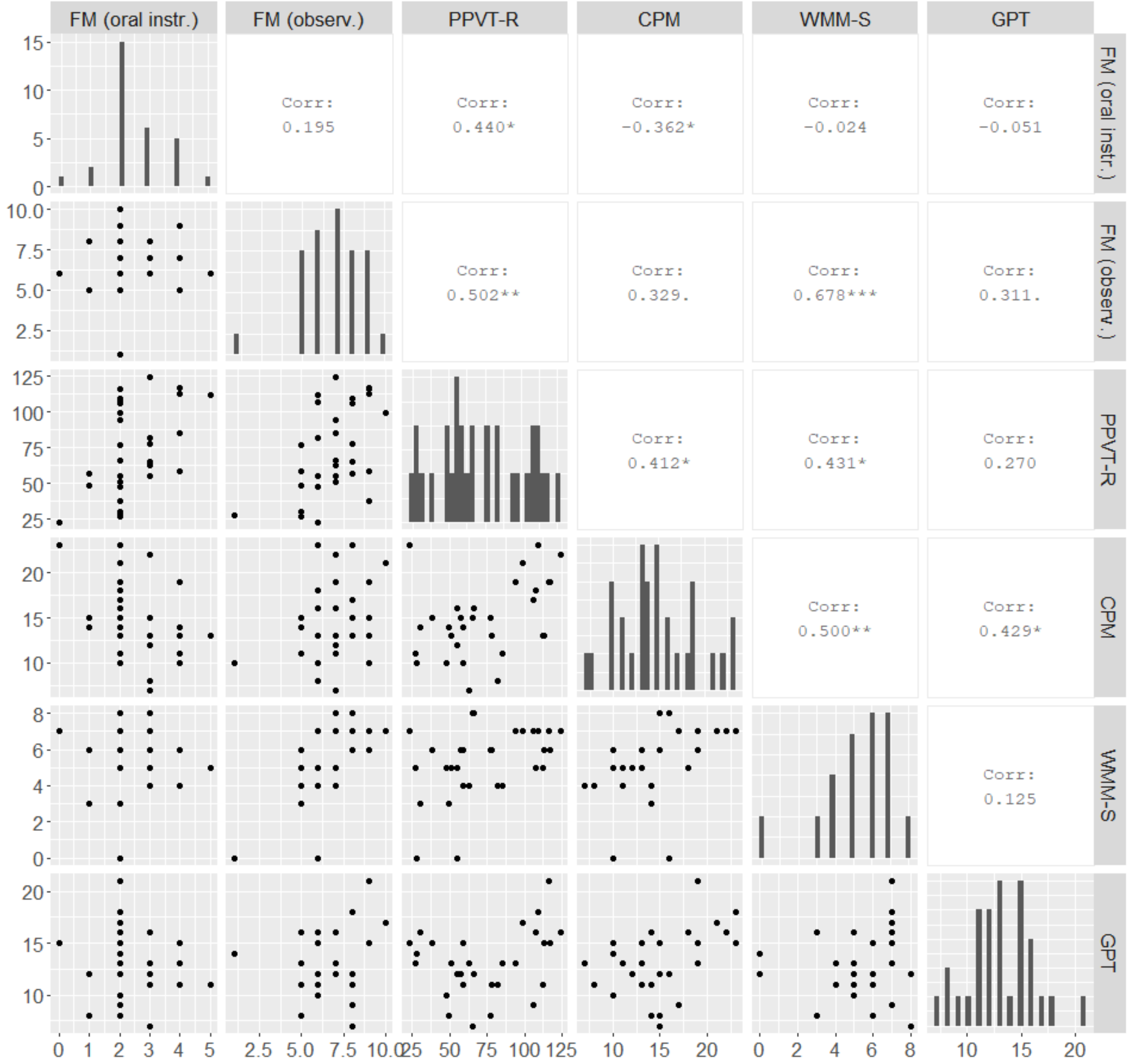

Supplementary Figure 4. Scatterplots, histograms, and correlations for all variables of interest in the Down syndrome group (N = 30). The correlations are not partialized by age because all participants in this group are adults.
